# Intraoperative surgical site infection control and prevention: a position paper and future addendum to WSES intra-abdominal infections guidelines

**DOI:** 10.1186/s13017-020-0288-4

**Published:** 2020-02-10

**Authors:** Belinda De Simone, Massimo Sartelli, Federico Coccolini, Chad G. Ball, Pietro Brambillasca, Massimo Chiarugi, Fabio Cesare Campanile, Gabriela Nita, Davide Corbella, Ari Leppaniemi, Elena Boschini, Ernest E. Moore, Walter Biffl, Andrew Peitzmann, Yoram Kluger, Michael Sugrue, Gustavo Fraga, Salomone Di Saverio, Dieter Weber, Boris Sakakushev, Osvaldo Chiara, Fikri M. Abu-Zidan, Richard ten Broek, Andrew W. Kirkpatrick, Imtiaz Wani, Raul Coimbra, Gian Luca Baiocchi, Micheal D. Kelly, Luca Ansaloni, Fausto Catena

**Affiliations:** 1 Department of General Surgery, Azienda USL-IRCSS di Reggio Emilia, Guastalla Hospital, Via Donatori di sangue 1, 42016 Guastalla, RE Italy; 2 Department of General Surgery, Macerata Hospital, 62100 Macerata, Italy; 3 General, Emergency and Trauma Surgery, Pisa University Hospital, 56124 Pisa, Italy; 4 Department of Surgery and Oncology, Hepatobiliary and Pancreatic Surgery, Trauma and Acute Care Surgery, University of Calgary Foothills Medical Center, Calgary, Alberta T2N 2T9 Canada; 5 Anesthesia and Critical Care Department, Papa Giovanni XXIII Hospital, P.zza OMS 1, 24128 Bergamo, Italy; 6 Emergency Surgery Unit and Trauma Center, Cisanello Hospital, Pisa, Italy; 7 Fabio Cesare Campanile, Ospedale Andosilla, Civita Castellana, Italy; 8 Unit of General Surgery, Castelnuovo ne’Monti Hospital, AUSL, Reggio Emilia, Italy; 9 Anesthesia and Critical Care Department, Papa Giovanni XXIII Hospital, P.zza OMS 1, 24128 Bergamo, Italy; 10 Abdominal Center, Helsinki University Hospital Meilahti, Helsinki, Finland; 11 Medical Library, Papa Giovanni XXIII Hospital, P.zza OMS 1, 24128 Bergamo, Italy; 12 Ernest E Moore Shock Trauma Center at Denver Health and University of Colorado, Denver, USA; 13 Trauma and Acute Care Surgery, Scripps memorial Hospital, La Jolla, CA USA; 14 Department of Surgery, University of Pittsburgh School of Medicine, Pittsburgh, USA; 15 Division of General Surgery, Rambam Health Care Campus, Haifa, Israel; 16 Department of Surgery, Letterkenny University Hospital and Donegal Clinical Research Academy, Letterkenny, Ireland; 17 Division of Trauma Surgery, School of Medical Sciences, University of Campinas, Campinas, SP Brazil; 18 Cambridge University Hospitals NHS Foundation Trust, Cambridge, UK; 19 Trauma and General Surgery, Royal Perth Hospital, Perth, Australia; 20 University Hospital St George First, Clinic of General Surgery, Plovdiv, Bulgaria; 21 State University of Milan, Acute Care Surgery Niguarda Hospital, Milan, Italy; 22 Department of Surgery, College of Medicine and Health Sciences, UAE University, Al-Ain, United Arab Emirates; 23 Radboud University Nijmegen, Nijmegen, The Netherlands; 24 Foothills Medical Centre and the University of Calgary, Calgary, AB Canada; 25 Department of Surgery, Sheri-Kashmir Institute of Medical Sciences, Srinagar, India; 26 Department of Surgery, UC San Diego Medical Center, San Diego, USA; 27 Department of Surgery, University of Brescia, Brescia, Italy; 28 Department of General Surgery, Albury Hospital, Albury, NSW 2640 Australia; 29 Department of Emergency and Trauma Surgery, Bufalini Hospital, 47521 Cesena, Italy; 30 Department of Emergency and Trauma Surgery, University Hospital of Parma, 43100 Parma, Italy

**Keywords:** Emergency, Surgical site infection, Prevention, Intra-abdominal infection, Operating room

## Abstract

**Background:**

Surgical site infections (SSI) represent a considerable burden for healthcare systems. They are largely preventable and multiple interventions have been proposed over past years in an attempt to prevent SSI.

We aim to provide a position paper on Operative Room (OR) prevention of SSI in patients presenting with intra-abdominal infection to be considered a future addendum to the well-known World Society of Emergency Surgery (WSES) Guidelines on the management of intra-abdominal infections.

**Methods:**

The literature was searched for focused publications on SSI until March 2019. Critical analysis and grading of the literature has been performed by a working group of experts; the literature review and the statements were evaluated by a Steering Committee of the WSES.

**Results:**

Wound protectors and antibacterial sutures seem to have effective roles to prevent SSI in intra-abdominal infections. The application of negative-pressure wound therapy in preventing SSI can be useful in reducing postoperative wound complications.

It is important to pursue normothermia with the available resources in the intraoperative period to decrease SSI rate.

The optimal knowledge of the pharmacokinetic/pharmacodynamic characteristics of antibiotics helps to decide when additional intraoperative antibiotic doses should be administered in patients with intra-abdominal infections undergoing emergency surgery to prevent SSI.

**Conclusions:**

The current position paper offers an extensive overview of the available evidence regarding surgical site infection control and prevention in patients having intra-abdominal infections.

## Background

Surgical site infections (SSI) are a common type of healthcare-associated infections and frequent complication of hospitalization, responsible for prolonged hospital stay, increased intensive care unit admissions, hospital readmissions after surgery, significantly increased costs (1300–5000 USD per SSI), and delays to adjuvant systemic therapy; they occur in 2 to 5% of patients undergoing surgery in the USA [1–3].

Approximately 160,000 to 300,000 SSI are diagnosed and treated every year and represent a considerable burden for healthcare systems in terms of re-operation, increased post-surgical pain, poor wound healing, prolonged hospital stay, cosmetic appearance, and decreased quality of life [4–7].

SSI has also been shown to be an independent risk factor in the development of incisional hernia [8].

The incidence of all types of SSI following abdominal surgery can reach 14% of all hospital-acquired infections and the most common form is the incisional superficial SSI, which is often the first to appear and is easy to diagnose [9].

While more data are available from Western healthcare settings, SSI was the leading cause of hospital-acquired infection in a systematic review of studies in low- and middle-income countries [10].

They also a result in deleterious softer endpoints such as patient psychosocial distress, loss of income, and decreased productivity [1–3].

Multiple interventions have been proposed and employed over the past decades in an attempt to prevent SSI. These include skin cleansing protocols, hair removal, the maintenance of intraoperative normothermia, the preoperative antimicrobial prophylaxis administration, the use of plastic adhesive skin barriers, the high flow oxygen supplementation, the wound protection, the sterility of instruments, the bowel preparation, the length of the incision, and the delayed primary incision closure [11–15].

The development of SSI is multifactorial, and it may be related to patient’s risk factors such as age, comorbidities, smoking habit, obesity, malnutrition, immunosuppression, malignancies, and the class of contamination of the wound [9, 16].

Emergency surgery is a risk factor for SSI because many strong risk factors for SSI such as contaminated and dirty wounds, prolonged duration of the operation, patient comorbidities, and high American Society of Anesthesiologists (ASA) score are commonly present in this type of surgery. For these reasons, the World Society of Emergency Surgery (WSES) developed a position paper for the prevention of SSI in the operative room (OR).

A panel of international experts discussed statements based on predetermined research questions and the results of related systematic literature reviews.

The literature search found few articles focused on SSI and emergency surgery; consequently, most of the reviewed studies considered the incidence of SSI in elective surgery because of the lack of valid data from an emergency setting. This is a consequence of the difficulty to conduct a good-quality study in an emergency environment: the workload is often intermittent and unpredictable, patient case-mix is heterogeneous with a wide variety of concomitant problems and severity of initial diagnosis; moreover, the emergency environment poses many barriers and obstacles to patient recruitment and data collection, and this has implications particularly for the staffing of prospective trials.

Considering all these limitations, we cannot ignore the potential benefit from using some devices and equipment or adopting some simple strategies in emergency surgery to decrease the incidence of SSI.

This position paper aims to provide recommendations on OR prevention of SSI in patients with intra-abdominal infections to be an addendum to the WSES Guidelines on the management of intra-abdominal infections.

## Materials and methods

In July 2018, the Scientific Board of the WSES, the President of the Society and the President of the 5th World Congress of the WSES decided to prepare a position paper on OR prevention of SSI in patients with intra-abdominal infections in the emergency setting.

The Presidents and ten members of the Scientific Secretariat (SS) agreed on 11 key topics to develop in the position paper (Table 1); nine international experts, members of the WSES Board, were chosen as Steering Committee (SC).

**Table 1:** Summary of statements

Main topics	Statements
1) *How to close a surgical incision?*	Statement 1.1: There is no significant difference in terms of SSI incidence and length of hospital stay between patients in which the skin is sutured by continuous versus interrupted stitches (GoR 1B) Statement 1.2: Superficial wound dehiscence is lower in subcuticular continuous suture versus interrupted stitches. (GoR 1B) Statement 1.3: The use of steri-strips doesn't reduce the incidence of SSI
2) *Coated sutures: are they useful?*	Statement 2: Triclosan-coated sutures significantly reduce SSI prevalence compared with the non-coated sutures (GoR1B)
3) *What is the role of intraoperative intraperitoneal irrigation vs topic wound lavage with antibiotic solutions to prevent surgical site infections?*	Statement 3: There are insufficient data to to support the role of intraperitoneal the role of intraperitoneal or topic wound irrigation with antibiotics in preventing SSI
4) *Could wound irrigation with saline and/or povidone iodine solution be useful to prevent surgical site infections?*	Statement 4: There are insufficient data to determine the role of saline or povidone solution irrigation of incisional wounds before closure to prevent SSI (GoR 2B).
5) *Are wound protector devices useful?*	Statement 5.1: The use of wound protectors has protective effects in reducing incisional SSI (GoR 1A); Statement 5.2: The use of dual-ring constructed wound protectors appears to be superior to single-ring devices in preventing SSI (GoR1B).
6) *Are sterile surgical drapes useful?*	Statement 6: There is no evidence that plastic adhesive incise drapes with or without antimicrobial properties are useful to decrease SSI (GoR 2C).
7) *To drain or not to drain in closing surgical incision?*	Statement 7: There are insufficient data to determine the role of the use of subcutaneous drainage of incisional wounds before closure to prevent SSI in high-risk patients (GoR 2B)
8) *When is double gloving recommended? When is changing gloves recommended during an operation?*	Statement 8.1: There are insufficient data to determine the role of double gloving to prevent SSI (GoR 2C). Statement 8.2: The mechanical resistance of latex gloves depends on the duration of wear. It may be beneficial for surgical team members and their protection to change gloves at certain intervals during surgery (GoR 2C).
9) *Is negative-pressure wound dressing useful to prevent surgical site infections?*	Statement 9: The application of negative-pressure wound therapy in preventing SSI may be effective in reducing postoperative wound complications and it may be an option especially in patients with a high risk of SSI (GoR 2C)
10) *Is intraoperative normothermia useful to prevent surgical site infections?*	Statement 10.1: Intraoperative normothermia decreases the rate of SSI (GoR 1A). Statement 10.2: The use of active warming devices in operating room is useful to keep normothermia and reduce SSI (GoR 1B)
11) *Is perioperative supplemental oxygen effective to reduce surgical site infections?*	Statement 11: Perioperative hyperoxygenation does not reduce SSI (GoR 2B)
12) *Leaving the skin open for delayed primary closure can reduce SSI?*	Statement 12.1: Delayed primary skin closure may reduce the incidence of SSI (GoR 2C) Statement 12.2: Delayed primary closure of a surgical incision is an option to take into consideration in contaminated abdominal surgeries, in patients with high risk of SSI (GoR 2C)
13) *When should additional antibiotic dose be administered intraoperatively?*	Statement 13: Optimal knowledge and use of the pharmacokinetic/pharmacodynamic characteristics of antibiotics are important to evaluate when additional antibiotic doses should be administered intraoperatively in patients with intra-abdominal infections undergoing emergency surgery (GoR 1C)

Each topic was developed by members of the SS: the SC and the Presidents supervised every step of literature search, selection, and the final work.

The SS provided the electronic search in PubMed and EMBASE databases, according to specific keywords for each question as you can see in the Appendix 1 without time or language restrictions.

Each expert followed the PRISMA methodology in the selection of papers to consider for review: meta-analyses of randomized controlled trials, randomized control trials, prospective studies, observational studies, large case series, and systematic reviews were included in this study.

Each SS member developed a focused draft and a variable number of statements. Each statement has been evaluated according to the Grading of Recommendations, Assessment, Development and Evaluation (GRADE) [17] summarized in Table 2.

**Table 2:** Grading of Recommendations, Assessment, Development and Evaluation (GRADE). *RCTs* randomized controlled trials

Grade of recommendation	Quality of supporting evidence	Implications
1A Strong recommendation, high-quality evidence	RCTs without important limitations or overwhelming evidence from observational studies	Strong recommendation, applies to most patients in most circumstances without reservation
1B Strong recommendation, moderate-quality evidence	RCTs with important limitations (inconsistent results, methodological flaws, indirect analyses or imprecise conclusions) or exceptionally strong evidence from observational studies	Strong recommendation, applies to most patients in most circumstances without reservation
1C Strong recommendation low-quality or very low-quality evidence	Observational studies or case series	Strong recommendation but subject to change when higher quality evidence becomes available
2A Weak recommendation high-quality evidence	RCTs without important limitations or overwhelming evidence from observational studies	Weak recommendation, the best action may differ depending on the patient, treatment circumstances, or social values
2B Weak recommendation moderate-quality evidence	RCTs with important limitations (inconsistent results, methodological flaws, indirect or imprecise) or exceptionally strong evidence from observational studies	Weak recommendation, the best action may differ depending on the patient, treatment circumstances, or social values
2C Weak recommendation low-quality or very low-quality evidence	Observational studies or case series	Very weak recommendation; alternative treatments may be equally reasonable and merit consideration

The provisional statements and the supporting literature were reviewed by all SS members and the Presidents, discussed with the SC members by email/call conferences and modified if necessary.

The designated member of SS presented the statements to SC along with the grade of recommendation (GoR) and the literature supporting each statement.

Clinicians and surgeons must be aware that the present position paper should be considered as an adjunctive tool for decision and management, but they do not substitute for the clinical judgment for individual patients.

## Results

### How to close a surgical incision?

#### Statement 1.1: There is no significant difference in terms of SSI incidence and length of hospital stay between patients in which the skin is sutured by continuous versus interrupted stitches (GoR 1B).

#### Statement 1.2: Superficial wound dehiscence is lower in subcuticular continuous suture versus interrupted stitches (GoR 1B).

#### Statement 1.3: The use of steri-strips or tissue adhesives doesn't reduce the incidence of SSI (GoR 1B).

The method of skin closure may have a role in preventing the development of SSI. Compared with interrupted sutures, continuous sutures can provide a better seal preventing the exogenous bacterial invasion of the surgical wound [16].

However, a continuous tightly pulled suture can strangulate the wound edges [18, 19].

Many published trials have demonstrated the benefit of skin closure by subcuticular interrupted sutures compared with conventional skin stapling in different surgical scenarios [9, 16, 17].

On the other hand, very few papers have been designed to investigate differences in the outcome when the skin is closed by continuous or by interrupted sutures.

In a Cochrane meta-analysis [19] published in 2014 and focused on the impact that different methods of skin closure could have on superficial SSI, superficial wound dehiscence, and length of hospital stay, only five RCTs comparing continuous versus interrupted sutures were identified. The five RCTs included a total of 827 participants undergoing abdominal or groin operations (non-obstetric surgery) [19–23]. Most of the enrolled patients were children or adolescents, and appendectomy was the most performed surgery.

Comparisons were made irrespectively of the material of the sutures. From this meta-analysis, no statistically significant differences were found between the two methods of suture regarding the prevalence of superficial SSI (RR 0.73; 95% CI 0.40 to 1.33) and length of hospital stay. However, a lower rate of superficial wound dehiscence was recorded in the continuous suture group (RR 0.08; 95%, CI 0.02 to 0.35).

It should be noted that in these trials the continuous skin suture groups received absorbable subcuticular sutures, while the interrupted skin suture groups received non-absorbable transcutaneous sutures. The non-absorbable sutures were removed 7 to 9 days after surgery, which is generally considered to be a suitable time for removal of sutures. The removal of sutures was not necessary for the absorbable subcuticular continuous suture group. The suture material used in the continuous suture groups was 4-0 poliglecaprone and 4-0 polyglactin [22, 23].

This kind of sutures retains approximately 50 to 75% of their original tensile strength after 1 week in situ. This extra support for the wound after 1 week may be the main reason for the difference between the continuous suture group and the interrupted suture group regarding the development of superficial wound dehiscence [19].

Conclusions of the meta-analysis were that superficial wound dehiscence may be reduced by using continuous subcuticular sutures and that continuous or interrupted skin closure does not have any impact on the development of superficial SSI and on the length of hospital stay. Due to the quality of the evidence, a high grade of uncertainty remains.

In addition to the abovementioned meta-analysis, only one study compared continuous versus interrupted skin suture for abdominal surgery in a non-intra-abdominal infection setting [24].

This review included 586 patients from a single Japanese institution to compare the incidence of incisional SSI after elective hepato-pancreatobiliary surgery (HPB) by different methods of skin closure. The study showed statistically significant efficacy of the subcuticular continuous sutures to prevent incisional SSI in patients undergoing HPB surgery (1.8% in the subcuticular continuous suture group and 10.0% in the stapling group, *P* < 0.01). However, the retrospective and single-institution design substantially affect the evidence of the results.

Many papers showing the benefits of subcuticular sutures versus stapling in terms of reduction of SSI and wound dehiscence are available from the literature, but unfortunately they were designed to compare interrupted rather than continuous subcuticular sutures versus stapling, or they merge continuous and interrupted techniques in a single group [9, 16, 25].

For these reasons, further well-designed RCTs with a low risk of bias should be conceived to establish which type of skin suturing provides better results.

A common practice in OR is to cover the closed wound with adhesive steri-strips.

Custis et al. [26] carried out a prospective study to assess whether the addition of adhesive strips to a wound closed with buried interrupted subcuticular sutures improves outcomes following wound closure. The study enrolled 45 patients and showed that there was no significant difference in the total patient assessment scale score between the combination closure (14.0 [7.6]) and sutures only (14.7 [7.6]) sides at 3 months (*P* = .39). There was also no significant difference between the two closure methods in terms of mean (SD) scar width (both methods, 1.1 [0.8] mm, *P* = .89) at follow-up. There was one case of wound dehiscence at a site that used adhesive strips and two cases at sites without adhesive strips. Three suture abscesses were documented at sites with adhesive strips and six at sites without adhesive strips. One patient had a spitting suture, which was not classified as an abscess; this event occurred at a site without adhesive strips. There were no documented infections, hematomas, or seromas. None of the adverse effects were statistically significant between study arms. The authors concluded that similar outcomes were observed whether or not adhesive strips were applied in addition to buried dermal sutures when performing cutaneous surgical procedures and that the use of adhesive strips cannot be recommended to improve cosmetic outcomes or reduce scar width.

An updated Cochrane review [27] was carried out to determine the effects of various tissue adhesives compared with conventional skin closure techniques for the closure of surgical wounds included 33 studies with a total of 2793 participants and demonstrated that there was low-quality evidence that sutures were significantly better than tissue adhesives for reducing the risk of wound breakdown (dehiscence; RR 3.35; 95% CI 1.53 to 7.33; 10 trials, 736 participants that contributed data to the meta-analysis). The number needed to treat for an additional harmful outcome was calculated as 43. For all other outcomes—infection, patient and operator satisfaction and cost—there was no evidence of a difference for either sutures or tissue adhesives. No evidence of differences was found between tissue adhesives and tapes for minimizing dehiscence, infection, patients’ assessment of cosmetic appearance, patient satisfaction, or surgeon satisfaction. The authors concluded that sutures are significantly better than tissue adhesives for minimizing dehiscence. In some cases, tissue adhesives may be quicker to apply than sutures.

### Coated sutures: are they useful?

#### Statement 2.: Triclosan-coated suture significantly reduces SSI prevalence compared with the non-coated sutures (GoR 1B).

Sutures with antimicrobial properties were developed to prevent microbial colonization of the suture material in operative incisions. Early studies showed a reduction of the number of bacteria in vitro and wound infections in animals using triclosan-coated sutures, and this effect was subsequently confirmed in clinical studies [28, 29]. Several novel antimicrobial coatings are now available, but still, no clinical studies have been done that compare the efficacy with non-coated sutures [30].

Wu et al. performed a systematic review to assess whether the use of antimicrobial-coated sutures is more effective in reducing the risk of SSI than the use of non-coated sutures.

Eighteen studies comparing triclosan-coated sutures vs non-coated sutures (13 randomized controlled studies and 5 observational studies) were included in the meta-analysis for a total of 7458 patients; all studies investigated triclosan-coated sutures and focused on adult patients, apart from one done in a pediatric population [31]. The meta-analysis of the data demonstrated that antimicrobial sutures significantly reduced SSI risk (for RCTs: OR 0.72, 95% CI 0.59–0.88, *P* = 0.001, I2 = 14%; for observational studies: OR 0.58, 95% CI 0.40–0.83, *P* = 0.003, I2 = 22%). Only Vicryl Plus vs Vicryl revealed consistent results in favor of antimicrobial sutures (for 7 RCTs: OR 0.62, 95% CI 0.44–0.88, *P* = 0.007, I2 = 3%; for 4 observational studies: OR 0.58, 95% CI 0.37–0.92, *P* = 0.02, I2 = 41%). Besides, the effect of antimicrobial coating was similar between different suture, wound (clean, clean-contaminated, and mixed), and procedure types (colorectal, cardio-vascular, head and neck, breast surgical procedures). Quality of RCT evidence was judged moderate, and observational studies’ evidence was judged of very low quality and many studies had conflicts of interest. The authors concluded that triclosan-coated sutures may reduce SSI risk.

Uchino et al. [32] have recently analyzed the efficacy of antimicrobial-coated sutures in preventing SSIs in digestive surgery. A total of 5188 patients in 15 studies were included, with 10 randomized controlled trials (RCT) and 5 observational studies (OBS). One study enrolled pediatric patients. The sutured surgical sites in the included studies were the abdominal fascia in 12 studies, the subcutaneous alone in 1 study, and unknown in 2 studies.

Regarding the types of surgeries represented, there were 9 colorectal surgeries, 4 mixed digestive surgeries, 1 gastric surgery, and 1 pancreaticoduodenectomy. The RCTs included 6 studies that performed surgeries limited to class 2 wounds or described the incidence distinct from the wound class. Only one study was performed during emergent surgeries and was limited to the dirty/infected wound classes. The remaining 3 studies were analyses conducted together with mixed wound classes. Regarding the suture materials in the RCTs, monofilament sutures were used in 4 RCTs, and poly-filament sutures were used in 4 RCTs. Two RCTs used mixed suture materials. In OBSs, nearly half of the participants had upper gastrointestinal surgery. The meta-analysis showed that in the 10 RCTs, the incidence rates of incisional SSIs were 160/1798 (8.9%) with coated sutures and 205/1690 (12.1%) with non-coated sutures. Overall, antimicrobial-coated sutures were superior for reducing the incidence of incisional SSI (RR 0.67, 95% CI 0.48–0.94, *P* = 0.02) in RCTs for digestive surgery with the mixed wound class and surgeries limited to a clean-contaminated wound (RR 0.66, 95% CI 0.44–0.98, *P* = 0.04). A superior effect of antimicrobial-coated sutures was found in 9 RCTs that involved only colorectal surgeries (RR 0.69, 95% CI 0.49–0.98, *P* = 0.04). The superior effect of antimicrobial-coated sutures was also found in OBSs (OR 0.4, 95% CI 0.3 to 0.54, *P* < 0.001). The mean hospital stay length was similar to coated or uncoated sutures in 5 RCTs involving colorectal surgery (mean difference (MD) − 5.00, 95% CI 16.68-6.69, *P* = 0.4) [32].

Guo et al. demonstrated that triclosan-coated sutures were associated with a lower risk of SSI than uncoated sutures across all surgeries (risk ratio [RR] 0.76, 95% confidence interval [CI] 0.65–0.88, *P* < 0.001). Similar proportions of patients experienced wound dehiscence with either type of suture (RR 0.97, 95% CI 0.49–1.89, *P* = 0.92). Subgroup analysis showed lower risk of SSI with triclosan-coated sutures in abdominal surgeries (RR 0.70, 95% CI 0.50–0.99, *P* = 0.04) and group with prophylactic antibiotic (RR 0.79, 95% CI 0.63–0.99, *P* = 0.04). However, such risk reduction was not observed in cardiac surgeries, breast surgeries, or the group without prophylactic antibiotics [33].

Henriksen et al. [34] in an overall comparison including both triclosan-coated Vicryl and PDS sutures for fascial closure, reported that triclosan-coated sutures were superior in reducing the rate of SSI (OR 0.67; CI 0.46–0.98). The majority of the studies included only elective surgery procedures. Four of these included only colorectal procedures, whereas Diener et al. [35] included all types of elective procedures through a midline laparotomy. Justinger et al. [36] included both elective and emergency laparotomies, whereas Ruiz-Tovar et al. [37] included only cases with fecal peritonitis and Mingmalairak et al. [38] studied patients undergoing open appendectomies. When evaluating PDS sutures separately, there was no effect of triclosan coating on the rate of SSI (OR 0.85; CI 0.61–1.17). After trial sequential analysis, authors concluded that triclosan-coated Vicryl sutures for abdominal fascial closure significantly decrease the risk of SSI and performing further RCTs will not change this outcome, but there was no effect on SSI rate with the use of triclosan-coated PDS sutures for abdominal fascial closure [34]. That means that PDS commonly used in abdominal surgery was not different.

Konstantelias et al. [39] analyzed 30 studies (19 randomized, 11 non-randomized; 15,385 procedures) giving evidence that triclosan-coated sutures were associated with a lower risk of SSIs (risk ratio [RR] = 0.68; 95% confidence interval [CI] 0.57–0.81). Triclosan-coated sutures were associated with a lower risk for SSIs in high-quality randomized studies (Jadad score 4 or 5). A lower risk for the development of SSIs based on wound classification was observed in clean, clean-contaminated, and contaminated but not for dirty procedures. No benefit was observed in specific types of surgery: colorectal, cardiac, lower limb vascular, or breast surgery.

A specific study on emergency surgery was also carried out confirming these findings [40].

### What is the role of intraoperative intraperitoneal irrigation vs topic wound lavage with antibiotic solutions to prevent surgical site infections?

#### Statement 3: There are insufficient data to support the role of intraperitoneal or topic wound irrigation with antibiotics in preventing SSI (GoR 2B).

Although intraoperative irrigation with antibiotic solutions has been suggested to be beneficial in the prevention of infections, no evidence-based results have been made available. The effectiveness of intra-abdominal lavage with antibiotic solutions on the prevention of postoperative SSI is controversial. Furthermore, issues about its safety need to be examined as well as local adverse effects (increased adhesion formation, postoperative pain), selection of resistant bacteria, and tissue toxicity.

The safety of the intraperitoneal administration of antibacterial agents during or after surgery as prophylaxis or treatment of infection has been investigated in a systematic review that included 29 RCTs and 50 observational studies [41].

The objective of this systematic review was to analyze perioperative intraperitoneal administration of antibacterial agents, to characterize the drugs used, and their safety profile. Administration of topical intraperitoneal antibiotics both during and after surgery was studied. Aminoglycosides, first- and second-generation cephalosporins, tetracyclines, and penicillins were most commonly administered intraperitoneally during or after surgery. The antibacterial agent was usually administered intraperitoneally as monotherapy. However, some studies administered combination regimens with heparin or with another antibacterial agent. The most frequent combination was aminoglycosides and lincosamides. Only a few and mild adverse events were reported and the authors concluded that antibacterial agents can safely be administered intraperitoneally. However, they acknowledged that in 43% of the included articles the adverse events were not reported while 41% of the studies specified that there were no adverse events related to the intraperitoneal administration of drugs. The most frequently reported adverse event was discomfort or pain during administration, especially with the use of oxytetracycline [41].

Animal data about the relationship between intraperitoneal antibiotics and adhesion development are conflicting [42–46].

In the experimental study conducted by Sortini et al. [43], the peritoneal lavage solution showing low adhesion formation and high survival rates was saline solution at 37 °C. In this study, lavage with antiseptics was associated with higher mortality (55–80% versus 0% for chlorhexidine–iodine solutions and saline solution, respectively, *P* < 0.001) but less adhesion formation (*P* < 0.001) as compared to saline solution. The use of antibiotic solutions was associated with 3% mortality in the treatment of peritonitis but with higher Zühlke scores and adhesion formation as compared to saline solution (*P* < 0.001).

According to these data, antiseptic solutions should not be recommended for peritoneal lavage.

Another experimental study was carried out to test the effectiveness of the intraperitoneal application of alternate antibiotics (Imipenem, ceftriaxone, and cefazolin) in an abdominal sepsis model. These data suggest that cephalosporins may be effective in preventing adhesion formation in septic abdomens compared to metronidazole [46].

Tetikcok et al. [47] have recently demonstrated that in rats, peritoneal lavage with prednisolone improved survival rates with increasing doses in abdominal sepsis. Abdominal lavage in rats was made using saline in group 1, equal volumes of cefazolin sodium in group 2, low-dose methylprednisolone (1 mg/kg) in group 3, and high-dose methylprednisolone (2 mg/kg) in group 4. The study showed that the mortality rate of the rats in group 2 was significantly higher than that in group 4, which had no mortality (*P* = 0.032). Although insignificant, the lowest mean value of IL-1β, IL-2, and TNF-α was in group 1, and the highest was in group 2. The lowest IL-4 level was in group 3, and the highest level was in group 2 (*P* = 0.41). Interleukin-10 levels were significantly lower in group 4 and higher in group 2 (*P* = 0.014). The administration of prednisolone in this abdominal sepsis model does not reflect a real-world situation; however, the administration of prednisolone alone helped to understand the effect of corticosteroids without masking the effects with antibiotics.

A 2017 Cochrane review included 36 studies (6163 participants) comparing the use of antibacterial irrigation with non-antibacterial irrigation [48]; authors reported a lower incidence of SSI in patients treated with antibacterial irrigation compared with non-antibacterial irrigation (RR 0.57, 95% CI 0.44 to 0.75; I2 = 53%; 30 studies, 5141 participants). This was low-certainty evidence downgraded once because 54% of the analysis weight was contributed by studies at high risk of bias in one or more domains, and once because publication bias was considered likely to have affected the result. Besides, the review pools together studies about intra-cavitary and wound irrigation, antibiotics, and antiseptics as antibacterial agents.

The possible benefit was present in each of the surgical contamination subgroups (clean versus clean-contaminated versus contaminated or dirty). The difference in adverse events, mortality, and abscess formation did not reach statistical significance. The hospital stay was reduced in the antibacterial irrigation group.

Concerning intraoperative wound irrigation, Mueller et al. in a meta-analysis of RCTs investigating the incidence of postoperative SSI after intraoperative irrigation of the surgical incision (after the closure of the fascia or peritoneum and before skin closure) performed a subgroup analysis comparing intraoperative wound irrigation with topical antibiotics vs saline solution irrigation. The study showed a significant reduction of postoperative SSI when antibiotic solution irrigation was used compared to both saline solution only irrigation and no irrigation.

The reported length of follow-up in the included trials was 30 days or more in only 21 out of 41 trials. The remaining trials reported follow-up times of as short as 5–10 days or did not specify the follow-up time at all. Besides, the number and frequency of follow-up visits varied largely, as did the type and blinding status of the primary outcome assessor [49].

However, the considerable risk for bias of all the included trials, their large heterogeneity, and the need to balance those findings against the risk of impaired wound healing and the potential increase of the bacterial resistance suggest caution in the clinical application of these results.

### Could wound irrigation with saline and/or povidone iodine solution be useful to prevent surgical site infection?

#### Statement 4.: There are insufficient data to determine the role of saline or povidone irrigation of incisional wounds before closure to prevent SSI (GoR 2B)

Intraoperative wound irrigation refers to the flow of a solution across the surface of an open wound. It is a widely practiced procedure and considered to help prevent SSI.

Among other benefits, wound irrigation is intended to physically remove foreign material, cellular debris, surface bacteria, and body fluids, to dilute possible contamination and to function as a local antibacterial agent when an antiseptic or antibiotic agent is used.

Wound irrigation must be vigorous enough to perform the above goals but gentle enough to avoid further tissue trauma or passage of bacteria and foreign material deeper into the wound. Practices vary depending on the patient population, the surface of the application, and the solution used.

On the other hand, vigorous irrigation may remove protective immunologic cells that are enable healing to progress through a natural series of processes, including inflammation and granulation, to final re-epithelialization and remodeling. Exposed subcutaneous tissue provides a favorable substratum for a wide variety of microorganisms to contaminate and colonize, and if the involved tissue is devitalized (e.g., ischemic, hypoxic, or necrotic) and the host immune response is compromised, the conditions become optimal for microbial growth [50]. A systematic review was carried out to investigate whether intraoperative wound irrigation (with or without active agents or pressured application) affects the incidence of SSI. Studies investigating the topical application of antibiotics or antiseptics (e.g., powder, gels, sponges) were not included.

Twenty-one RCTs were identified comparing wound irrigation with no wound irrigation in patients undergoing various surgical procedures, and the results were substantially heterogeneous [51]

Saline irrigation was not effective in reducing SSIs [52]. However, when the saline was applied with a syringe to generate some pressure [53], a reduction in the risk of SSI compared with no irrigation was shown in one study (OR 0.35; 95% CI 0.19–0.65; *P* = 0.0009). This benefit also was demonstrated when pulse pressure irrigation with saline was compared with normal saline irrigation in a meta-analysis of two RCTs [54, 55] (OR 0.30; 95% CI 0.08–0.86; *P* = 0.0003).

In the same meta-analysis, a low quality of evidence demonstrated a statistically significant benefit for incisional wound irrigation with an aqueous povidone iodine solution in clean and clean-contaminated wounds (OR 0.31; 95% CI 0.13–0.73; *P* = 0.007); 50 fewer SSI per 1000 procedures (from 19 fewer to 64 fewer) [51].

The 2017 Cochrane review comparing antibacterial irrigation with non-antibacterial irrigation (36 studies, 6163 participants), the largest meta-analysis published, reported a lower incidence of SSI in participants treated with antibacterial irrigation compared with non-antibacterial irrigation (RR 0.57, 95% CI 0.44 to 0.75; I2 = 53%; 30 studies, 5141 participants) but evidence are of low certainty [48].

Therefore, where a possible difference in the incidence of SSI was identified (in comparisons of antibacterial and non-antibacterial interventions, and pulsatile versus standard methods), these should be considered in the context of uncertainty, particularly given the possibility of publication bias for the comparison of antibacterial and non-antibacterial interventions.

Clinicians should also consider whether the evidence is relevant to the surgical populations (wound classification and setting) under consideration.

### Are wound protector devices useful? (Table 3)

#### Statement 5.1: The use of wound protectors has protective effects in reducing incisional SSI (GoR 1A);

#### Statement 5.2: The use of dual-ring constructed wound protectors appears to be superior to single-ring devices in preventing SSI (GoR 1B).

Wound protector devices (alternatively called “wound guards” or “wound retractors”) have been increasingly used in the effort to reduce SSI rates. These devices form a physical barrier between the wound edges and the contaminated surgical field. More specifically, the impervious plastic barrier prevents both endogenous and exogenous pathogens from imbedding themselves within the wound (skin, fat, fascia, peritoneum). This mechanism, in conjunction with maintaining wound humidity and reducing direct physical trauma from fixed retractors, is believed to reduce the risk of incisional SSI. It must be noted however that some bacterial invasion could occur immediately before the insertion, or more likely after the removal of the wound protector itself. There are two widely available forms: a single ring that lies within the abdominal cavity connected to a protective drape that extends outward, or two rings that are connected cylindrically by impenetrable plastic with one ring inside the wound and the other secured on the outside [64].

**Table 3:** The effectiveness of wound protectors [57–63]: characteristics of the studies included in the review. *RCT*: randomized controlled trial; *SSI*: surgical site infection; *PCT*: prospective controlled trial; *GoR*: grade of recommendation

Author and year of publication	Type of study	Number of patients	Outcomes	GoR
Pinkney TD et al. 2013 [56]	Multicenter RCT	760	Wound edge protection devices do not reduce the rate of surgical site infection in patients undergoing laparotomy, and therefore their routine use for this role cannot be recommended.	1A
Gheorghe A et al. 2012 [57]	Systematic review and meta-analysis of 2 PCT + 10 RCT	1933	Wound edge protectors may be efficient in reducing SSI rates in patients undergoing open abdominal surgery	1B
Edwards JP et al. 2012 [58]	Meta-analysis of 6 RCT	1008	Wound protectors reduce rates of SSI after gastrointestinal and biliary surgery	1A
Mihaljevic AL et al. 2015 [59]	Systematic review and meta-analysis of 16 RCT	3695	Wound edge protectors significantly reduce the rate of surgical site infections in open abdominal surgery	1B
Zhang MX et al. 2015 [60]	Systematic review and meta-analysis of 11 RCT	2344	Wound edge protector reduces the incidence of SSI in patients receiving laparotomies, especially in the circumstance of dual-ring type and in contaminated incisions. In order to fully assess the effectiveness of WEP, large-scale and well-designed RCTs are still needed in the future.	1B
Kang SI et al. 2018 [61]	Systematic review and meta-analysis of 14 RCT	2684	Potentially significant benefit from impervious plastic wound protector use, greater protective effect in using dual-ring protector than a single ring	1A
Sajid MS et al. 2017 [62]	Systematic review and meta-analysis of 18 RCT	3808	Wound edge protector is associated with reduced incidence of overall SSI in clean-contaminated and contaminated wounds	1B
Bressan AK et al. 2018 [63]	RCT	107	Among adult patients with intrabiliary stents, the use of a dual-ring wound protector during pancreaticoduodenectomy significantly reduces the risk of incisional SSI.	1A

The ROSSINI trial [56] is a multicenter observer-blinded RCT carried up to determine the clinical effectiveness of wound edge protection device (the device used was the 3 M Steri-Drape Wound Edge Protector) in reducing surgical site infection after abdominal surgery, enrolling 760 patients with 382 patients assigned to the device group and 378 to the control group, reported that a total of 184 patients experienced surgical site infection within 30 days of surgery, 91/369 (24.7%) in the device group and 93/366 (25.4%) in the control group (odds ratio 0.97, 95% confidence interval 0.69 to 1.36; *P* = 0.85). In the secondary analyses, no subgroup could be identified in which there was evidence of clinical benefit associated with the use of the device. The authors concluded that wound edge protection devices cannot be recommended to reduce the rate of SSI in patients undergoing laparotomy.

Gheorghe et al. cost-effectiveness analysis suggests that the use of wound protector devices for SSI reduction cannot be justified and should be discontinued [64].

Previously, in 2012, Gheorghe et al. [57] reviewed 12 studies (2 prospective controlled studies +10 RCTs) reporting primary data from 1933 patients. The quality assessment found all of them to be at considerable risk of bias. An exploratory meta-analysis was performed to provide a quantitative indication of the wound edge protector device effect. The pooled risk ratio under a random-effects model was 0.60 (95% confidence interval, 0.41–0.86), indicating a potentially significant benefit from the use of the dispositive. No indications of significant between-study heterogeneity or publication bias, respectively, were identified.

In 2012, Edwards et al. [58] analyzed 6 RCTs for a total of 1008 patients were included. They reported that the use of a wound protector was associated with a significant decrease in SSI (RR = 0.55, 95% CI 0.31–0.98, *P* = 0.04). Data showed also a nonsignificant trend toward greater protective effect in studies using a dual-ring protector (RR = 0.31, 95% CI 0.14–0.67, *P* = 0.003), rather than a single-ring protector (RR = 0.83, 95% CI 0.38–1.83, *P* = 0.64).

To assess these controversial results, several meta-analyses have been published looking at the effectiveness of wound protectors in preventing SSIs in abdominal surgeries.

In 2015, Mihaljevic et al. [59] analyzed 16 RCTs including 3695 patients investigating wound edge protectors published between 1972 and 2014. Data reported that wound edge protectors significantly reduced the rate of surgical site infections (risk ratio 0.65; 95%CI, 0.51–0.83; *P* = 0.0007; *I*^2^ 2 = 52%). A similar effect size was found in the subgroup of patients undergoing colorectal surgery (risk ratio 0.65; 95%CI, 0.44–0.97; *P* = 0.04; *I*^2^ 2 = 56%). Of the two common types of wound protectors, double-ring devices were found to exhibit a greater protective effect (risk ratio 0.29; 95%CI, 0.15–0.55) than single-ring devices (risk ratio 0.71; 95%CI, 0.54–0.92), but this might largely be due to the lower quality of available data for double-ring devices. Exploratory subgroup analyses for the degree of contamination showed a larger protective effect in contaminated cases (0.44; 95%CI, 0.28–0.67; *P* = 0.0002, *I*^2^ 2 = 23%) than in clean-contaminated surgeries (0.72, 95%CI, 0.57–0.91; *P* = 0.005; *I*^2^ 2 = 46%) and a strong effect on the reduction of superficial surgical site infections (risk ratio 0.45; 95%CI, 0.24–0.82; *P* = 0.001; *I*^2^ 2 = 72%) [59].

Zhang et al. reviewed 11 RCTs including 2344 patients. In particular, 6 trials (1589 patients) testing the single-ring design wound edge protector did not show a statistically significant reduction in SSI of laparotomy (RR 0.76, 95% CI 0.51–1.12). Pooled analysis of the five trials (755 patients) that tested the effect of dual-ring wound protector on SSI showed a significant reduction (RR 0.29, 95% CI 0.15–0.55). The combined data of the 11 trials favored the wound edge protector effect (RR 0.58, 95% CI 0.39–0.87). Analysis adjusted by the degrees of contamination revealed that wound protector device is effective in reducing the incidence of SSI after laparotomy incision contamination (RR 0.43, 0.26–0.72) but failed to demonstrate such effect in clean/contaminated and dirty incisions (RR 0.72, 95% CI 0.43–1.21; RR 0.82, 95% CI 0.43–1.55, respectively) [60]

More specifically, two extremely recent systematic reviews that evaluated 2684-patient and 3808-patient RCTs respectively once again confirm this observation.

The first from Kang et al. [61] identified and analyzed 14 randomized controlled trials with a total of 2684 patients. The pooled risk ratio under a random-effects model was 0.70 (95% confidence interval, 0.51-0.96; I2, 56.8%), indicating a potentially significant benefit from impervious plastic wound protector use. There was a significant trend toward greater protective effect in studies using a dual-ring protector (relative risk = 0.31; 95% confidence interval, 0.15–0.58), rather than a single-ring protector (relative risk = 0.84; 95% confidence interval, 0.71–1.00). There was no significant between-study heterogeneity or publication bias.

The second from Said et al. [62] analyzed 18 RCTs and demonstrated that wound edge protector is associated with the reduced incidence of overall SSI (OR 0.59; 95% CI 0.43–0.81; *z* = 3.30; *P* < 0.001) and superficial SSI (OR 0.42; 95% CI 0.18–0.95; *z* = 2.09; *P* < 0.04). In addition, it also successfully reduced the risk of SSI in clean-contaminated wounds (OR 0.67; 95% CI 0.46–0.98; *z* = 2.06; *P* < 0.04) as well as in contaminated wounds (OR 0.24; 95% CI 0.12–0.49; *z* = 3.96; *P* < 0.0001). The reported overall reduction in SSI was substantial in both reviews (OR = 0.70 and 0.59 respectively).

When superficial (wound) SSI is the focus of the analysis, there is a further reduction in the postoperative rate (OR = 0.42). Furthermore, these trends appear to extend to both clean-contaminated and contaminated wounds (OR = 0.67 and 0.24 respectively). While these comprehensive reviews and statistical analyses are compelling, they omit a single large recent RCT that evaluated the role of wound protectors in high-risk non-colorectal scenarios (i.e., pancreaticoduodenectomies (PD) following preoperative insertion of biliary stents for obstruction). This study including a total of 107 patients reported a significant reduction in the incidence of incisional SSI in the wound protector group (21.1% vs 44.0%; relative risk reduction 52%; *P* = 0.010). Patients with completed PD had a decrease in incisional SSI with the use of the wound protector compared with those undergoing palliative operations (27.3% vs 48.7%; *P* = 0.04). Multivariate analysis did not identify any significant modifying factor relationships (estimated blood loss, duration of surgery, hospital site, etc.) (*P* > 0.05) [63].

While the utility of wound protectors is clear, the superior mechanical configuration of these devices remains debated. More specifically, both single-ring (with or without large adhesive drape components) and dual-ring modalities (internal and external ring connected by impervious plastic) are currently available. Two high-quality analyses [61, 62] have both noted a strong trend toward a greater protective effect with dual-ring variants when compared to devices constructed with a single external ring and associated semi-adhesive drape. It is also interesting to note that among this level 1 RCT data, there is a clear modifying effect of the publication year. In other words, as time has progressed in the study of wound protectors (and therefore the evaluation of more diverse surgical subgroups), their protective effect has become increasingly evident.

In clinical practice, the only possible barrier to the routine use of these types of devices is cost and availability. A possible solution to decrease cost is to reserve wound protectors for high-risk patients or dirty surgical incisions to reduce SSI and equate costs related to wound protectors and hospitalization(s).

### Are adhesive sterile surgical incise drapes useful?

#### Statement 6.1: There is no evidence that plastic adhesive drapes with or without antimicrobial properties are useful to decrease SSI (GoR 2C).

Adhesive plastic incise drapes are used on a patient’s skin after surgical site preparation, with or without antimicrobial impregnation, and the surgeon performs the incision of the drape and the skin simultaneously. There are conflicting recommendations on the use of plastic adhesive drapes, mainly discouraging their use.

In 2015, the fourth update of the Cochrane review carried out to investigate the advantages about using plastic adhesive drapes to protect the wound from organisms that may be present on the surrounding skin during surgery, analyzed 5 studies with a total 3082 participants comparing plastic adhesive drapes with no drapes and 2 studies involving 1113 participants comparing iodine-impregnated adhesive drapes with no drapes. A significantly higher proportion of patients in the adhesive drape group developed a surgical site infection when compared with no drapes (risk ratio (RR) 1.23, 95% confidence interval (CI) 1.02 to 1.48, *P* = 0.03). Iodine-impregnated adhesive drapes did not affect the surgical site infection rate (RR 1.03, 95% CI 0.06 to 1.66, *P* = 0.89). The length of hospital stay was similar in the adhesive drape and non-adhesive drape groups. There was no evidence from the 7 trials that plastic adhesive drapes reduce surgical site infection rates and some evidence that they increase infection rates [65].

In 2016, Allegranzi et al. analyzed 4 studies (one RCT, one quasi-RCT, and two observational studies) comparing adhesive iodine-impregnated incise drapes with no drapes and showed no difference in the SSI risk (RCTs: OR 2·62; 0·68–10·04; observational studies: OR 0·49; 0·16–1·49). Similarly, a meta-analysis of two RCTs comparing non-impregnated adhesive incise drapes to no drapes showed no difference in the SSI risk (OR 1·10; 0·68–1·78) [66].

Recently, Rezapoor et al. carried out a prospective, randomized clinical trial to evaluate the efficacy of iodophor-impregnated adhesive drapes for reducing bacterial contamination and counts at the incision site during hip surgery. The study enrolled 101 patients undergoing open joint preservation procedure of the hip. Half the patients had the adhesive drape applied to the skin before incision, while the remainder underwent the same surgery without a drape. Culture swabs were taken from the surgical site at 5 points (pre skin preparation, after skin preparation, post-incision, before subcutaneous closure, before dressing application) and sent for culture and colony counts. After surgery, 12.0% of incisions with adhesive drapes and 27.4% without adhesive drapes were positive for bacterial colonization. It appears that the iodophor-impregnated adhesive draping significantly reduces bacterial colonization of the incision [67].

Recently, Zarei et al. have conducted a quasi-experimental study with non-equivalent control group design enrolling 88 patients who were the candidate for lumbar spine surgery in the elective operating room to investigate the effect of the incise drape on the rate of bacterial contamination of surgical wound, and they concluded that the use of ID is unable to reduce surgical wound bacterial contamination in clean lumbar spine surgery [68].

### To drain or not to drain in closing surgical incision?

#### Statement 7.1: There are insufficient data to determine the role of subcutaneous drainage of incisional wounds before closure to prevent SSI in high-risk patients (GoR 2B).

Evidence regarding the utility of subcutaneous drains in preventing incisional SSI are controversial.

The presence of fluid collection between the skin sutures and underlying fascia is thought to increase the risk for SSIs, as it can provide a medium for bacterial growth. The concept of subcutaneous drainage is to remove these fluids before they become infected, resulting in a reduction of SSI.

Recently, several studies have examined suctioning/active drainage systems as a means to prevent SSI in digestive surgery, but the utility of these systems is still controversial [69, 70].

Fuji et al. assessed the efficiency of subcutaneous drains for high-risk patients undergoing colorectal surgery, including patients with thick subcutaneous fat tissue and those undergoing emergency operations. They enrolled in their 79 high-risk patients for SSI. The overall incidence of incisional SSI was 27.8%. The incidences of incisional SSI in these cases with or without a subcutaneous drain were 14.3% and 38.6%, respectively. The authors concluded that subcutaneous drains are effective for preventing incisional SSI in patients with thick subcutaneous fat in colorectal surgery [71].

In 2013, Kosins et al. [72] reviewed and analyzed 52 randomized controlled trials with a total of 6930 operations aimed to determine the evidenced-based value of prophylactic drainage of subcutaneous wounds in surgery. Subgroups were determined by specific surgical procedures or characteristics (cesarean delivery, abdominal wound, breast reduction, breast biopsy, femoral wound, axillary lymph node dissection, hip and knee arthroplasty, obesity, and clean-contaminated wound). There were 3495 operations in the drain group and 3435 in the no-drain group. Prophylactic subcutaneous drainage offered a statistically significant advantage only for the prevention of hematomas in breast biopsy procedures and the prevention of seromas in axillary node dissections. In all other procedures studied, drainage did not offer an advantage.

The authors concluded that drain placement following a surgical procedure is the surgeon’s choice and can be based on multiple factors beyond the type of procedure being performed or the patient’s body habitus [72].

All the previous studies assessed the usefulness of active-suctioning subcutaneous drain in a closed surgical wound. Numata et al. [73] decided to evaluate the efficacy of a passive drainage system for preventing surgical site infections during major colorectal surgery, enrolling 246 (124 underwent passive drainage, and 122 underwent no drainage) patients who underwent major colorectal surgery. Patients were randomly assigned to receive subcutaneous passive drainage or no drainage. The primary outcome measured was the incidence of superficial SSI. The secondary outcomes measured were the development of hematomas, seromas, and wound dehiscence.

They reported a significant difference in the incidence of superficial SSIs between patients assigned to the passive drainage and no drainage groups (3.2% vs 9.8%, respectively, *P* = 0.041). There were no cases that developed a hematoma, seroma, or wound dehiscence in either group. The authors concluded that subcutaneous passive drainage provides benefits over no drainage in patients undergoing major colorectal surgery.

The benefit of subcutaneous drainage was studied also in ileostomy closure that is in a dirty surgical field; after having conducted an RCT, Lauscher et al. [74] were able to affirm that the omission of subcutaneous suction drains is not inferior to the use of subcutaneous suction drains after ileostomy reversal in terms of length of hospital stay, surgical site infections, and hematomas/seromas.

In another RCT, the rate of SSI appeared to be reduced with subcutaneous suction drains in open abdominal surgery, but the authors concluded that prospective randomized larger-scale studies should be performed to confirm data [75].

Recently, Watanabe et al. [76] decided to evaluate the effects of subcutaneous closed-suction Blake drain for preventing SSIs after colorectal surgery performing an RCT, enrolling 240 patients. The incidence of incisional SSI was 8.7% in the overall patients. The incidence of incisional SSI was 12.8% in the control arm and 4.5% in the subcutaneous drainage arm. They reported a significant reduction of the incidence of SSI in the subcutaneous drainage arm than in the control arm (*P* = 0.025). Logistic regression analysis demonstrated that thickness of subcutaneous fat > 3.0 cm, forced expiratory volume in 1 s as percent of forced vital capacity (FEV1.0%) > 70%, and subcutaneous drain were independent predictors of postoperative incisional SSIs (*P* = 0.008, *P* = 0.004, and *P* = 0.017, respectively). The authors affirmed that a subcutaneous Blake drain is beneficial for preventing incisional SSIs in patients undergoing colorectal surgery [76].

Manzoor et al. [77] after reviewing the literature to assess the evidence on the efficacy of subcutaneous wound drainage in reducing SSI concluded that not all patients will benefit from subcutaneous drainage. Subcutaneous wound drainage seems to be useful in patients with high risk to develop an SSI including patients who are obese and/or have contaminated wounds but in clean and clean-contaminated surgical wounds, it remains a surgeon’s choice [77].

### When is double gloving recommended? When is changing gloves recommended during an operation?

#### Statement 8.1: There are insufficient data to determine the role of double gloving to prevent SSI (GoR 2B).

#### Statement 8.2: The mechanical resistance of latex gloves depends on the duration of wear. It may be beneficial for surgical team members and their protection to change gloves at certain intervals during surgery [GoR 2C].

Surgical gloves are an important physical barrier between the surgical staff and the patient. They enable the prevention of transmission of microorganisms in both directions, from the surgeons’ hands to the patient.

The integrity of gloves depends on the duration of wearing, the role within the surgical team, and the type of surgery performed.

Their use since the beginning was a barrier against infections. With the recognition of HIV infection and the associated concerns about transmission of HBV and hepatitis C virus in the operating room during the 1980s and early 1990s, considerable interest emerged in the provision of better protection of the hands for surgical personnel [78].

The intact surgical glove is the most important barrier to the bi-directional migration of microorganisms between the hands of the members of a surgical team and the patient. Several studies have shown that undetected perforations of surgical gloves are common and that the frequency of such defects increases with the duration of glove wear. The risk of glove defects is related to the type of surgery being done, ranging from 7% in urologic surgery to 65% in cardiothoracic surgery [78, 79].

Various measures have been developed to reduce the risk of surgical site contamination with microorganisms originating from the surgeon’s hands.

Standard practice for decreasing the microbial bio-burden on the hands of surgeons and other surgical team members is preoperative surgical hand disinfection with an antimicrobial soap (surgical scrub) or an alcohol-based hand disinfectant (surgical rub). Preoperative surgical hand disinfection can reduce, but not eradicate, the resident flora on the surgeon’s hands. Because of the re-growth of skin flora during a surgical procedure, original levels of skin flora on a surgeon’s hands can be re-established within 3–6 h, depending on the formulation of the product used to disinfect the hands [78].

A novel sterile antimicrobial surgical glove, featuring a proprietary complex coating with 14 ingredients and chlorhexidine as an active antimicrobial ingredient on its inner surface, has been developed to reduce the risk of contamination of the surgical site in the event of a glove breach. Further clinical studies are needed to confirm this concept [79].

Double gloving has been demonstrated to reduce blood contact with the hands of the operating team. Quebbeman and colleagues noted a nearly 90% reduction in hand exposure to blood with double gloving in a prospective, randomized trial [80]. Wearing two pairs of latex gloves significantly reduces the number of perforations to the innermost glove. This evidence comes from trials undertaken in “low-risk” surgical specialties. Wearing two pairs of latex gloves does not cause the glove wearer to sustain more perforations to their outermost glove. Wearing double latex indicator gloves enables the glove wearer to detect perforations to the outermost glove more easily than when wearing double latex gloves. However wearing a double latex indicator system will not assist with the detection of perforations to the innermost glove, nor reduce the number of perforations to either the outermost or the innermost glove. There is no direct evidence that additional glove protection worn by the surgical team reduces surgical site infections in patients; however, the most important published review has insufficient power for this outcome [81]..

The adequate protection, however, requires that the glove material remain intact. The electrical conductivity, insulation, and mechanical resistance of glove latex depend on the duration of wear. Latex is subject to hydration; 30 min of surgical use was associated with measurable hydration of glove latex and a statistically significant loss of electrical and mechanical resistance, with rupture load decreasing by 24% [82].

Parteke et al. prospectively collected 898 consecutive pairs of used surgical gloves over 9 months in a single institution and reported that wearing gloves for 90 min or less resulted in microperforations in 46 (15.4%) of 299 pairs of gloves, whereas wearing gloves for 91–150 min resulted in perforation of 54 (18.1%) of 299 pairs, and 71 of (23.7%) of 300 pairs were perforated when the duration of wear was longer than 150 min (*P* = .05). Because of the increase in the rate of microperforation over time, authors recommended that surgeons, first assistants, and surgical nurses directly assisting in the operating field change gloves after 90 min of surgery [83].

Several studies demonstrated that the occurrence of microperforations in surgical gloves increases over time.

Even in orthopedic surgery, surgical gloves should be changed when they are excessively contaminated with surgical fluids and the surgeon and first assistant should also change their outer gloves at an average of every 90 min [84].

Glove perforation rates are high in open abdominal surgery; considering data available, it may be beneficial for surgical team members to change gloves at certain intervals during surgery or use indicator glove systems [84].

### Is negative-pressure wound dressing useful to prevent surgical site infections? (Table 4)

#### Statement 9: The application of negative-pressure wound therapy in preventing SSI may be effective in reducing postoperative wound complications and it may be an option, especially in patients with a high risk of SSI. (GoR 2C).

Gomoll et al. [93] first reported the application of negative-pressure wound therapy in closed incisions (cINPT), and their outcomes showed that its use for treating closed incisions in orthopedic surgery can reduce the incidence of SSI.

**Table 4:** Negative wound dressing in preventing SSI: characteristics of the studies included in the review [85–96]. *SSI* surgical site infection, *RCT* randomized controlled trial, *GoR* grade of recommendation, *NPWT* negative-pressure wound therapy, *LOS* lengh of hospital stay

Author and year of publication	Type of study	Number of patients	Outcomes	GoR
Sandy-Hodgetts K et al. (2015) [88]	Systematic review and meta-analysis of 8 (RCT, pseudo-randomized trials, quasi-experimental studies, prospective and retrospective cohort studies, case control studies, and analytical cross sectional studies)	1277	NPWT in preference to standard postoperative dressings may be considered for closed surgical incisions in adults assessed as high-risk for SSI; further research is needed (level 1 studies—RCT) on patients identified as “at risk” in the preoperative period.	2C
Strugala V et al. 2017 [89]	Meta-analysis of 10 RCT + 6 prospective observational trials	1863	The significant reduction in SSI, wound dehiscence, and LOS on the basis of pooled data shows a benefit of the PICO single-use NPWT system compared with standard care in closed surgical incisions.	1A
Sahebally SM et al. 2018 [90]	Systematic review and meta-analysis of 9 studies (3 RCT and 2 prospective and 4 retrospective studies)	1266	Application of NPWT on closed laparotomy wounds in general and colorectal surgery is associated with reduced SSI rates but similar rates of seroma and wound dehiscence compared with conventional nonpressure dressings.	2C
Webster J et al. 2019 [94]	Cochrain systematic review (30 interventional studies)	2957	uncertainty remains about whether NPWT compared with a standard dressing reduces or increases the incidence of important outcomes such as mortality, dehiscence, seroma, or if it increases costs. Given the cost and widespread use of NPWT for SSI prophylaxis, there is an urgent need for larger, well-designed and well-conducted trials to evaluate the effects of newer NPWT products designed for use on clean, closed surgical incisions. Such trials should initially focus on wounds that may be difficult to heal, such as sternal wounds or incisions on obese patients.	2C
Katsuki Danno et al. 2018 [95]	Prospective study	28	The use of NPWT is an effective measure for preventing SSI in patients undergoing abdominal surgery for peritonitis caused by lower-gastrointestinal perforation.	2C
Lozano-Balderas G et al. 2017 [96]	Prospective randomized study	81	Statistical significance was found between infection rates of the vacuum-assisted group and the other two groups (primary closure and delayed primary closure). The infection rate in contaminated/dirty-infected laparotomy wounds decreases from 37 and 17% with primary and delayed primary closures, respectively, to 0% with vacuum-assisted systems.	1C

A subsequent series of reports [85–87] confirmed the effectiveness of cINPT in reducing SSI.

In 2015, Sandy-Hodgetts et al. [88] decided to conduct a systematic review and meta-analysis of all papers available from 1990 to 2013 evaluating the effectiveness of cINPT in preventing postoperative surgical wound complications. Eight studies were included in the review. Meta-analyses revealed a statistically significant difference in favor of the use of cINPT as compared with standard surgical dressings in managing SSI, but conflicting results were found for wound dehiscence and seroma. Considering the small number of studies included and that most of them were retrospective comparative cohort in design, authors could not recommend cINPT to prevent SSI even if the study demonstrated an association between the use of cINPT and reduction of SSI.

A more recent meta-analysis by Strugala et al. [89] investigated the effectiveness of prophylactic use of a specific design of cINPT device on surgical site complications. The authors considered all articles comparing the specific single-use cINPT device (PICO) with standard care for SSI in closed surgical wounds. Ten randomized and 6 observational studies were selected with a total of 1863 patients (2202 incisions) included. The randomized studies reported a significant reduction in SSI rate of 51% from 9.7 to 4.8% with cINPT intervention (RR 0.49 [95% CI 0.34–0.69] *P* < 0.0001). The observational studies assessed a reduction in SSI rate of 67% from 22.5 to 7.4% with cINPT (RR 0.32 [95% CI 0.18–0.55] *P* < 0.0001). Pooling all the data, there was a significant reduction in SSI of 58% from 12.5 to 5.2% with cINPT (RR 0.43 [95% CI 0.32–0.57] *P* < 0.0001) regardless of the type of surgery (orthopedic, abdominal, colorectal, or cesarean section), although the numbers needed to treat were lower in operations with higher frequencies of complications. Furthermore, meta-analysis showed a significant reduction in dehiscence from 17.4 to 12.8% with cINPT (RR 0.71 [95% CI 0.54–0.92] *P* < 0.01) and in-hospital length of stay by cINPT (− 0.47 days [95% CI − 0.71 to − 0.23] *P* < 0.0001).

Another meta-analysis carried out by Sahebally et al. [90] in 2018 evaluated the association of prophylactic cINPT with SSI rates in general and colorectal surgery in elective and emergency settings.

Three randomized trials and 2 prospective and 4 retrospective studies were selected for the meta-analysis, involving 1187 patients with 1189 incisions. The authors found significant clinical and methodologic heterogeneity among the studies. On random-effects analysis, cINPT was associated with a significantly lower rate of SSI compared with standard dressings (pooled odds ratio [OR], 0.25; 95% CI, 0.12–0.52; *P* < .001) but no difference in rates of seroma (pooled OR, 0.38; 95% CI, 0.12–1.23; *P* = .11) or wound dehiscence (pooled OR, 2.03; 95% CI, 0.61–6.78; *P* = 0.25). On sensitivity analysis, focusing solely on colorectal procedures, cINPT significantly reduced SSI rates (pooled OR, 0.16; 95% CI, 0.07–0.36; *P* < .001). Thus, this study demonstrated that the application of cINPT on closed laparotomy wounds in general and in colorectal surgery is associated with reduced SSI rates but no different significant rates of seroma and wound dehiscence compared with traditional dressings.

Readership expressed some criticisms about the clinical value of these outcomes considering the high level of statistical heterogeneity associated with the included studies in the discussion and the necessity for randomized controlled trials before recommending the application of cINPT in clinical practice.

Uncertainty in the indications for the use of cINPT had been reported in 2012 [91] and then confirmed in 2014 [92] and the updated 2019 [94] version of the Cochrane systematic review. In the last systematic review, despite the addition of 25 trials, the authors judged the evidence to be low or very low certainty for all outcomes.

The study involved 2957 participants (30 intervention trials and two economic studies nested in trials). Surgeries included abdominal and colorectal (*n* = 5); cesarean sections (*n* = 5); knee or hip arthroplasties (*n* = 5); groin surgery (*n* = 5); fractures (*n* = 5); laparotomy (*n* = 1); vascular surgery (*n* = 1); sternotomy (*n* = 1); breast reduction mammoplasty (*n* = 1); and mixed (*n* = 1). Webster et al. showed uncertainty about whether cINPT compared with a standard dressing reduces or increases the incidence of important outcomes such as mortality, dehiscence, and seroma or if it increases costs. Given the cost and widespread use of cINPT for SSI prophylaxis, authors claimed an urgent need for larger, well-designed and well-conducted trials to evaluate the effects of newer cINPT products designed for use on clean, closed surgical incisions.

Several studies investigated the role of cINPT in contaminated and dirty surgical wounds.

Danno et al. [95] prospectively included in their study 28 patients undergoing abdominal surgery for peritonitis caused by a lower-gastrointestinal perforation. They compared data from this group with a 19 patients historical control group who had undergone primary suturing for managing peritonitis incisions for a lower-gastrointestinal perforation. Authors reported a significant association between the SSI incidence and the type of incision management (10.7% with cINPT and delayed closure vs. 63.2% with primary suturing; *P* < 0.001); no significant difference between the groups in the length of the hospital stay (22 days for cINPT and delayed closure vs. 27 days for primary suturing; *P* = 0.45) was found.

Therefore, the association of cINPT and delayed closure of the abdominal wall is an effective method to prevent SSI.

A Spanish group [96] decided to compare outcomes about three techniques used for wound management after laparotomy in contaminated and dirty/infected wounds: the primary, delayed primary, and vacuum-assisted closures in terms of SSI. Eighty-one patients undergone laparotomy with Class III or IV surgical wounds were enrolled in a three-arm randomized prospective study. Twenty-seven patients received primary closure, 29 delayed primary closure, and 25 vacuum-assisted closure, with no exclusions for analysis. Surgical site infection was present in 10 (37%) patients treated with primary closure, 5 (17%) with primary delayed closure, and 0 (0%) patients receiving vacuum-assisted closure. Statistical significance was found between infection rates of the vacuum-assisted group and the other two groups. No significant difference was found between the primary and primary delayed closure groups. The infection rate in contaminated/dirty-infected laparotomy wounds decreases from 37 and 17% with primary and delayed closures, respectively, to 0% with vacuum-assisted systems [96]. We have to consider that in this study the number of patients is very small for each group.

Several studies evaluated the cost-utility of cINPT in preventing SSIs compared to standard dressings and demonstrated that the use of closed-incision negative-pressure therapy is cost-saving following the closure of abdominal incisions in high-risk patients [97–99].

Furthermore, to obviate the high costs related to current equipment for cINPT, more cost-effective alternatives were developed using standard gauze sealed with an occlusive dressing and wall suction. Several studies comparing both methods of treatment appear to be similarly effective for reducing wound surface area and volume [94, 100, 101].

### Is intraoperative normothermia useful to prevent surgical site infections?

#### Statement 10.1: Intraoperative normothermia decreases the rate of SSI (GoR 1A).

#### Statement 10.2: The use of active warming devices in operating room is useful to keep normothermia and reduce SSI (GoR 1B).

Core body temperature is kept in a narrow range by several mechanisms, namely heat genesis and thermal insulation (mainly vasoconstriction or dilatation). This balance is greatly challenged during major surgery. On the one hand, surgery may imply exposure of large surface areas with consequent loss of heat and fluids. On the other hand, anesthesia disrupts the temperature setpoint (i.e., a lower than usual temperature triggers an adaptive reflex as shivering or metabolic thermogenesis) and can increase heat loss by vasodilatation [102]. Animal studies have shown that hypothermia increases complications such as infection, myocardial infarction, and coagulation derangements. Perioperative hypothermia can increase SSI due to its reflex vasoconstriction and mediated local immunosuppression. Vasoconstriction reduces partial oxygen pressure which lowers resistance to infections in animal models [103].

Perioperative normothermia has been addressed by several studies, papers, and meta-analysis. Considering only RCTs, the subsequent comparisons, but not limited to them, have been evaluated: head-to-head RCTs of one active warming device vs another, different extension of the active warming period through the perioperative one, active warming device vs no warming, warming of fluids and or insufflation gases during laparoscopic vs no active warming. We decided to focus on RCTs comparing interventions aimed at preventing hypothermia vs a control group where no such an intervention was implemented (a placebo group), the outcome was the incidence of SSI. Four relevant papers were analyzed [104, 105]. All of them dealt with an active body warming device against the placebo.

Kurz et al. [105] in 1996 randomized 200 patients scheduled for major abdominal contaminated surgery to receive active body surface warming by a forced-air warmer device. The incidence of SSI was 6/104 in the intervention group and 18/96 in the control one (*P* = 0.009).

Melling et al. [106] in 2001 randomized 421 patients scheduled for clean surgery into three arms placebo, local warming (non-contact, radiant heat dressing), and systemic warming (forced-air warming device). Pooling the data of the two intervention groups, the incidence of SSI was 19/139 in the placebo group vs 13/277 in the intervention group (*P* = 0.001).

Pu et al. [107] in 2014 randomized 110 patients scheduled for laparoscopic gastrointestinal procedure into placebo group vs systemic warming (disposable underbody warming blanket with reusable forced-air warming system). The incidence of SSI was 0 in both the intervention and control groups.

Yi et al. [104] in 2018 randomized, in an open-label, pilot study 62 patients scheduled for open thoracic or hip replacement surgery to systemic warming (forced-air warming device) vs control (quilt). The incidence of SSI was 0/32 in the control group and 3/30 in the warming group (*P* = 0.238).

The effectiveness of temperature measurement in preventing SSIs has been assessed in a large cohort 2013 study in the colonic surgery population [108]. Several meta-analyses have been published on the topic. A recent Cochrane review from Madrid et al. [106] reviewed the literature and found a significant decrease in SSI after the implementation of an active warming intervention (risk ratio (RR) 0.36, 95% confidence interval (CI) 0.20 to 0.66; *P* = 0.0008; *I*^2^ = 0%); the studies were rated of fair quality. Another meta-analysis reached the same conclusions [106]. There exists little debate around the effectiveness of reducing SSI by keeping the patients normothermic throughout the perioperative period. Four RCTs [100–103] and at least two meta-analyses [109, 110] confirm this risk reduction. It seems unlikely that other RCTs comparing a device to keep normothermia will be compared with a placebo group as this recommendation has been implemented in several national and international guidelines [111–114]. The last two RCTs [104, 107] with a real placebo group have been carried out in a nation where it is not common practice to warm patients during surgery. Those studies [100, 103] were meant to be pilot studies to assess the feasibility of forced-air warming in that context.

The two open questions are which device and/or strategy should be used and when (only intraoperative or intraoperative and pre- and/or postoperative?). There are three main devices to warm up the patients: forced-air warming (so far the most studied and used worldwide), resistive polymer fabric warming, and circulatory warming systems using a closed fluid circuit. The use of radiant heating systems is considered feasible only during pediatric procedures. On the other side, other strategies have been implemented to reduce heat loss and prevent hypothermia (e.g., warm iv infusion, warm irrigation fluids or gases for pneumoperitoneum during laparoscopic, preoperative infusion of nutrients to increase metabolic rate and protein turn-over, reflective blankets). A thorough evaluation of those questions is outside the statement. The majority of those studies has as main outcome the achievement of normothermia and were not powered enough to detect a difference in SSI. To date, Madrid et al. [109] evaluated in their meta-analysis the studies comparing head-to-head the different modality to warm up the patients and found no differences in SSI incidence. The main concern is the use of forced-air warming devices in surgery where air-borne pathogens are a major threat to orthopedic prosthesis surgery. In this particular scenario, the surgery takes place under the condition of ultra clean ventilation, at least in affluent countries, and it is known that forced-air disrupt the laminar flow and increases a load of bacteria at the operation site (in lab models). The bacterial load is the main risk factor for prosthesis colonization [115]. A systematic review is available but results are inconclusive [116]. Anyway, this hypothesis has not been formally tested in an adequately powered RCT.

The timing of warming has been evaluated in several papers. Pre-emptive warming plus intraoperative warming has shown better results in providing normothermia than intraoperative warming alone in small RCTs [117–119] and in a systematic meta-analysis [120]. Heterogeneity between the studies is high as well as the results from the single trials and the meta-analysis was not conclusive.

Several guidelines from national and international institutions stated in favor of achieving normothermia in the perioperative period to reduce the incidence of SSI [111–114].

### Is perioperative supplemental oxygen effective to reduce SSI?

#### Statement 11: Perioperative hyperoxygenation does not reduce SSI (GoR 2B).

The most important defense against SSI is oxidative killing by neutrophils, and molecular oxygen is the substrate of the process. The easiest way to increase tissue oxygenation is to increase inspired oxygen. For example, intraoperative tissue oxygen partial pressure is typically about 6.6 kPa in patients given 30% inspired oxygen and about13.3 kPa in those given 80% inspired oxygen [121].

Despite some early evidence [121], there have since been conflicting results from numerous randomized clinical trials.

Two well-conducted randomized trials (*n* = 500 and *n* = 300) [121, 122], a smaller trial [123] and a registry analysis [124], suggested that supplemental oxygen (80% vs 30%) halved infection risk, supporting the role of supplemental oxygen in reducing the risk of SSI. However, other studies have not been able to confirm this.

The PROXI trial [125], that is a large, multicenter, randomized trial involving 1400 patients undergoing abdominal surgery, found no evidence of any beneficial effect of supplemental oxygen; in fact, SSI occurred in 131 of 685 patients (19%) receiving 80% oxygen and in 141 of 701 (20%) receiving 30% oxygen [odds ratio 0.94 (95% confidence interval 0.72–1.22), *P* = 0.64]. Indeed, a long-term follow-up study (median 2.3 years after surgery) found poorer survival in the supplemental oxygen group [126].

Another recently published randomized, blinded trial including 400 patients [127] tested the hypothesis that extending intraoperative supplemental oxygen 12 to 16 h into the postoperative period reduces the risk of SSI and healing-related complications in the morbidly obese patients and reported no benefit of supplemental oxygen.

In 2018, Cohen et al. [128] published a meta-analysis including 26 trials with a total of 14,710 patients, to investigate the effect. The RR [95%CI] for wound infection was 0.81 [0.70, 0.94] in the high vs. low inspired oxygen groups. The effect remained significant in colorectal patients (10,469 patients), 0.79 [0.66, 0.96], but not in other patients (4,241 patients), 0.86 [0.69, 1.09]. When restricting the analysis to studies with low risk of bias, either by strict inclusion criteria (5047 patients) or by researchers’ judgment (12,547 patients), no significant benefit remained: 0.84 [0.67, 1.06] and 0.89 [0.76, 1.05], respectively. The authors concluded that meta-analysis of the most reliable studies does not suggest that supplemental oxygen substantively reduces wound infection risk when considering all available data, but more research is needed to fully answer this question.

Whether supplemental oxygen, which is inexpensive and easy to provide, reduces infection risk, thus remains in dispute.

### Leaving the skin open for delayed primary closure can reduce SSI?

#### Statement 12.1: Delayed primary skin closure may reduce the incidence of SSI (GoR2C).

#### Statement 12.2: Delayed primary closure of a surgical incision is an option to take into consideration in contaminated abdominal surgeries in high-risk patients (GoR 2C).

Delayed primary closure of dirty wounds has been widely practiced in war surgery; it is a procedure which aims to reduce the rate of SSI by suturing a wound later after proper dressing, considering the fundamental principles of decreasing bacterial inoculums and potentiating local wound resistance from increasing wound oxygenation and blood supply from developing granulation tissue. It was first applied to traumatic wounds and later was more widely applied to various types of operations with the demonstration of good efficacy [129–131].

These results were mainly from observational studies that may be prone to selection and confounding biases.

Besides, the delayed primary closure also has its disadvantages including pain from routine dressing, the necessity for later wound suturing, and increase the cost of treatments [129–132].

In 2013, Bhangu et al. [132] decided to determine using meta-analysis whether delayed primary skin closure of contaminated and dirty abdominal incisions reduces the rate of SSI compared with primary skin closure.

The authors included in the final analysis 8 studies randomizing 623 patients with contaminated or dirty abdominal wounds to either delayed primary skin closure or primary closure. The most common diagnosis was appendicitis (77.4%), followed by perforated abdominal viscus (11.5%), ileostomy closure (6.5%), trauma (2.7%), and intra-abdominal abscess/other peritonitis (1.9%). The time to the first review for delayed primary skin closure was provided at between 2 and 5 days postoperatively. All studies were found to be at high risk of bias, with marked deficiencies in study design and outcome assessment. When SSI was assessed across all studies using a fixed-effect model, delayed primary skin closure significantly reduced the chance of SSI (odds ratio, 0.65; 95% CI, 0.40–0.93; *P* = .02). However, heterogeneity was high (72%), and using a random-effects model, the effect was no longer significant (odds ratio, 0.65; 95% CI, 0.25–1.64; *P* = .36).

The authors concluded that delayed primary skin closure may reduce the rate of SSI, but current trials fail to provide definitive evidence.

In 2014, Siribumrungwong et al. [133] decided to investigate the same topic carrying out a systematic review and meta-analysis to compare SSI between delayed primary and primary wound closure in complicated appendicitis and other contaminated abdominal wounds. Eight studies were considered for meta-analysis: 5 studies were done in complicated appendicitis, 2 with mixed complicated appendicitis and other types of abdominal operation, and 1 with ileostomy closure. Most studies (75%) had a high risk of bias in sequence generation and allocation concealment. Among 6 RCTs of complicated appendicitis that underwent open appendectomy, the SSI between primary closure and delayed primary closure were not significantly different with a risk ratio of 0.89 (95% CI, 0.46, 1.73). Delayed primary closure had significantly 1.6 days (95% CI: 1.41, 1.79) longer length of stay than primary closure.

Based on a small number of studies with low-quality, a meta-analysis suggested there might be no advantage of delayed primary closure over primary closure in reducing SSI in complicated appendicitis.

After this meta-analysis, Siribumrungwong et al. [134] carried out a multicenter randomized controlled trial to compare superficial SSI rates between delayed primary wound closure and primary wound closure for complicated appendicitis.

The study enrolled and randomized 300 and 298 patients with gangrenous and ruptured appendicitis to primary closure and delayed primary closure (at postoperative days 3–5) groups.

The superficial SSI rate was lower in the primary closure than in delayed primary closure groups [i.e., 7.3% (95% confidence interval 4.4, 10.3) vs 10% (95% CI 6.6, 13.3)] with a risk difference (RD) of − 2.7% (− 7.1%, 1.9%), but this RD was not significant. Postoperative pain, length of stay, recovery times, and quality of life were nonsignificantly different with corresponding RDs of 0.3 (− 2.5, 3.0), − 0.1 (− 0.5, 0.3), − 0.2 (− 0.8, 0.4), and 0.02 (− 0.01, 0.04), respectively. However, costs for primary closure were 2083 (1410, 2756) cheaper than DPC ($60 USD).

The authors showed that superficial SSI rates for the primary closure group were slightly lower than the delayed group, even if there is no statistical significance. Costs were significantly lower for the primary closure group.

Recently, Tang et al. [135] published a meta-analysis about the benefits of a delayed primary closure over primary closure of a surgical incision in contaminated abdominal surgery.

Of the 12 studies included in the analysis, 5 were from third world countries (i.e., India and Pakistan), and all of these demonstrated an improvement in the SSI rate with delayed primary closure. When the fixed-effect model was used, compared with primary closure, SSI was significantly reduced in delayed primary closure with a risk ratio of 0.64 (0.51–0.79) (*P* < 0.0001), and a significant difference in LOS between delayed primary closure and primary closure was also identified with a mean difference of 0.39 (0.17–0.60) (*P* = 0.0004). Although the random-effect model was used, no significant difference in SSI between delayed and primary closure was observed with a risk ratio of 0.65 (0.38–1.12) (*P* = 0.12), and no significant difference in LOS with a mean difference of 1.19 (− 1.03 to 3.41) (*P* = 0.29).

The authors suggested that delayed primary closure may be the preferable choice in contaminated abdominal surgeries, especially in patients with a high risk of infection, and particularly in resource-constrained environments, even if more high-quality studies are needed to provide clear evidence.

### When should additional antibiotic doses be administered intraoperatively?

#### Statement 13: Optimal knowledge and use of the pharmacokinetic/pharmacodynamic characteristics of antibiotics are important to evaluate when additional antibiotic doses should be administered intraoperatively in patients with intra-abdominal infections undergoing emergency surgery (GoR 1C).

Optimal use of the pharmacokinetic/pharmacodynamic characteristics of antibiotics is helpful to evaluate when additional antibiotic doses should be administered intraoperatively in patients with intra-abdominal infections undergoing emergency surgery.

Antibiotics should be used after a treatable intra-abdominal infection (IAI) has been recognized or there is a high degree of suspicion of infection. Initial antimicrobial therapy for patients with IAI should be prompt because especially critically ill patients need immediate treatment. It may be interesting to evaluate when additional antibiotic doses should be administered intraoperatively in patients with intra-abdominal infections undergoing emergency surgery.

To define how to administrate antibiotics in patients with IAIs, it is necessary to know the pharmacokinetic/pharmacodynamic relationship of antibiotics. Knowledge of the pharmacokinetic and pharmacodynamic antibiotic properties may provide a more rational determination of optimal dosing regimens in terms of the dose and the dosing interval [136].

Antibiotic pharmacodynamics integrates the complex relationship between organism susceptibility and patient pharmacokinetics. Pharmacokinetics describes the fundamental processes of absorption, distribution, metabolism, and elimination and the resulting concentration-versus-time profile of an agent administered in vivo. The achievement of appropriate target site concentrations of antibiotics is essential to eradicate the pathogens [136]. Suboptimal target site concentrations may have important clinical implications and may explain therapeutic failures, in particular, for bacteria for which in vitro MICs are high. During the operation, target site concentrations should remain steadily optimal.

Dosing frequency is related to the concept of time-dependent versus concentration-dependent killing. Beta-lactam agents exhibit time-dependent activity and exert optimal bactericidal activity when drug concentrations are maintained above the MIC [137]. Therefore, the serum concentration must exceed the MIC for the appropriate duration of the dosing interval. Higher-frequency dosing, prolonged infusions, and continuous infusions have been utilized to achieve this effect. It is well known that for beta-lactams, prolonged or continuous infusions have been advocated to maximize the time that the drug concentration exceeds the MIC, whereas high peak concentrations are not beneficial. This concept should be extended also to patients undergoing an emergency operation and higher-frequency dosing, prolonged infusions, and continuous infusions should be suggested also in the operatory room.

In contrast, antibiotics such as aminoglycosides exhibit concentration-dependent activity and should be administered in a once-daily manner (or with the least possible number of daily administrations) to achieve high peak plasma concentrations [137].

With these agents, the peak serum concentration, and not the time the concentration remains above the MIC, is more closely associated with efficacy. In these patients, additional doses are not necessary during operation.

## Conclusions

We conceived this position paper to offer an extensive overview of available evidence regarding OR prevention of surgical site infection in emergency surgery as a potential addendum to WSES guidelines on the management of intra-abdominal infections.

The use of triclosan-coated suture significantly reduces SSI prevalence compared with the non-coated sutures.

The use of wound protectors has protective effects in reducing incisional SSI, in particular, the use of dual-ring constructed wound protectors appears to be superior to single-ring devices in preventing SSI.

The application of negative-pressure wound therapy in preventing SSI may be effective in reducing postoperative wound complications and it may be an option to take into consideration especially in patients with a high risk of infection.

Intraoperative normothermia decreases the rate of SSI, and the use of active warming devices in the operating room is useful to keep normothermia.

Perioperative supplemental oxygenation does not reduce SSI.

There is no strong evidence that delayed primary skin closure may reduce the incidence of SSI but it may be a valid option to primary skin closure in highly contaminated or “dirty” abdominal operations, especially in patients at high risk of infection.

The optimal knowledge and use of the pharmacokinetic/pharmacodynamic characteristics of antibiotics are important to evaluate when additional antibiotic doses should be administered intraoperatively in patients with intra-abdominal infections undergoing emergency surgery.

## Data Availability

Not applicable.

## Appendix 1

### Key words’ list for literature searching:

- “surgical incision” and “closure”“suture” and “surgical site infection”
- “irrigation” and “incisional wound”;
- “wound protector” and “surgical site infection”;
- “dual ring” and “wound protector” and “wound infection”;
- “incisional drape” and “wound infection”;
- “drainage” and “subcutaneous” and “surgical incision”;
- “gloves” and “surgical site infection”;
- “negative pressure wound therapy”and wound infection” and surgical incision”;
- “normothermia” and “surgical site infection” and warming device”;
- “antibiotics” and “surgical wound infection” and “prevention”;
- “hyperoxia/hyperoxigenation”and “surgical site infection”;
- “timing skin closure” and “early” and “delayed” and “wound infection” and “dirty surgical incision”.

## Abbreviations

cINPT: Closed-incision negative-pressure therapy
NPWT: Negative-pressure wound therapy
OBS: Observational trial(s)
OR: Operating room
RCT: Randomized controlled trial(s)
SC: Steering committee
SS: Scientific secretary
SSI: Surgical site infection(s)
