# Correction to: Intraoperative surgical site infection control and prevention: a position paper and future addendum to WSES intra-abdominal infections guidelines

**DOI:** 10.1186/s13017-021-00361-4

**Published:** 2021-04-14

**Authors:** Belinda De Simone, Massimo Sartelli, Federico Coccolini, Chad G. Ball, Pietro Brambillasca, Massimo Chiarugi, Fabio Cesare Campanile, Gabriela Nita, Davide Corbella, Ari Leppaniemi, Elena Boschini, Ernest E. Moore, Walter Biffl, Andrew Peitzmann, Yoram Kluger, Michael Sugrue, Gustavo Fraga, Salomone Di Saverio, Dieter Weber, Boris Sakakushev, Osvaldo Chiara, Fikri M. Abu-Zidan, Richard ten Broek, Andrew W. Kirkpatrick, Imtiaz Wani, Raul Coimbra, Gian Luca Baiocchi, Micheal D. Kelly, Luca Ansaloni, Fausto Catena

**Affiliations:** 1 Department of General Surgery, Azienda USL-IRCSS di Reggio Emilia, Guastalla Hospital, Via Donatori di sangue 1, 42016 Guastalla, RE Italy; 2 Department of General Surgery, Macerata Hospital, 62100 Macerata, Italy; 3 General, Emergency and Trauma Surgery, Pisa University Hospital, 56124 Pisa, Italy; 4 Department of Surgery and Oncology, Hepatobiliary and Pancreatic Surgery, Trauma and Acute Care Surgery, University of Calgary Foothills Medical Center, Calgary, Alberta T2N 2 T9 Canada; 5 Anesthesia and Critical Care Department, Papa Giovanni XXIII Hospital, P.zza OMS 1, 24128 Bergamo, Italy; 6 Emergency Surgery Unit and Trauma Center, Cisanello Hospital, Pisa, Italy; 7 Fabio Cesare Campanile, Ospedale Andosilla, Civita Castellana, Italy; 8 Unit of General Surgery, Castelnuovo ne’Monti Hospital, AUSL, Reggio Emilia, Italy; 9 Anesthesia and Critical Care Department, Papa Giovanni XXIII Hospital, P.zza OMS 1, 24128 Bergamo, Italy; 10 Abdominal Center, Helsinki University Hospital Meilahti, Helsinki, Finland; 11 Medical Library, Papa Giovanni XXIII Hospital, P.zza OMS 1, 24128 Bergamo, Italy; 12 Ernest E Moore Shock Trauma Center at Denver Health and University of Colorado, Denver, USA; 13 Trauma and Acute Care Surgery, Scripps memorial Hospital, La Jolla, CA USA; 14 Department of Surgery, University of Pittsburgh School of Medicine, Pittsburgh, USA; 15 Division of General Surgery, Rambam Health Care Campus, Haifa, Israel; 16 Department of Surgery, Letterkenny University Hospital and Donegal Clinical Research Academy, Letterkenny, Ireland; 17 Division of Trauma Surgery, School of Medical Sciences, University of Campinas, Campinas, SP Brazil; 18 Cambridge University Hospitals NHS Foundation Trust, Cambridge, UK; 19 Trauma and General Surgery, Royal Perth Hospital, Perth, Australia; 20 Clinic of General Surgery, University Hospital St George First, Plovdiv, Bulgaria; 21 Acute Care Surgery Niguarda Hospital, State University of Milan, Milan, Italy; 22 Department of Surgery, College of Medicine and Health Sciences, UAE University, Al-Ain, United Arab Emirates; 23 Radboud University Nijmegen, Nijmegen, The Netherlands; 24 Foothills Medical Centre and the University of Calgary, Calgary, AB Canada; 25 Department of Minimal Access and General Surgery, Government Gousia Hospital, Sringar, Kashmir India; 26 Department of Surgery, UC San Diego Medical Center, San Diego, USA; 27 Department of Surgery, University of Brescia, Brescia, Italy; 28 Department of General Surgery, Albury Hospital, Albury, NSW 2640 Australia; 29 Department of Emergency and Trauma Surgery, Bufalini Hospital, 47521 Cesena, Italy; 30 Department of Emergency and Trauma Surgery, University Hospital of Parma, 43100 Parma, Italy

**Correction to: World J Emerg Surg 15, 10 (2020)**

**https://doi.org/10.1186/s13017-020-0288-4**

The original article [1] contained an incorrect affiliation for co-author, Imtiaz Wani. This has since been corrected.
